#  Risk factors for ectopic pregnancy in Germany: a retrospective study of 100,197 patients

**DOI:** 10.3205/000260

**Published:** 2017-12-19

**Authors:** Louis Jacob, Matthias Kalder, Karel Kostev

**Affiliations:** 1 Faculty of Medicine, University of Paris 5, Paris, France; 2 Department of Gynecology and Obstetrics, Philipps-University Marburg, Marburg, Germany; 3 Epidemiology, QuintilesIMS, Frankfurt, Germany

**Keywords:** ectopic pregnancy, risk factors, Germany, retrospective study

## Abstract

**Aim:** The goal of this study was to identify potential risk factors for ectopic pregnancy in women followed in German gynecological practices.

**Methods:** The present study included pregnant women diagnosed with ectopic pregnancy and pregnant women without ectopic pregnancy followed in 262 gynecological practices between January 2012 and December 2016. The effects of demographic and clinical variables on the risk of developing ectopic pregnancy were estimated using a multivariate logistic regression model.

**Results:** This study included 3,003 women with ectopic pregnancy and 97,194 women without ectopic pregnancy. The mean age was 31.4 years (SD=5.9 years) in ectopic pregnancy patients and 31.1 years (SD=5.6 years) in non-ectopic pregnancy patients. Women aged 36–40 (OR=1.12) and 41–45 years (OR=1.46) were at a higher risk of ectopic pregnancy than women aged 31–35 years. Prior ectopic pregnancy was strongly associated with a risk of recurring ectopic pregnancy (OR=8.17). Prior genital surgery (OR=2.67), endometriosis (OR=1.51), and eight other gynecological diseases were also positively associated with ectopic pregnancy (ORs ranging from 1.19 to 2.06). Finally, there was a 1.80-fold increase in women previously diagnosed with psychiatric disorders.

**Conclusions:** Prior ectopic pregnancy and prior genital surgery were strongly associated with ectopic pregnancy in women followed in German gynecological practices. Psychiatric diseases had an additional impact on the risk of ectopic pregnancy.

## Zusammenfassung

**Ziel:** Das Ziel dieser Studie war es, potentielle Risikofaktoren für eine Extrauteringravidität bei in deutschen gynäkologischen Praxen behandelten Frauen zu identifizieren.

**Methodik:** Die vorliegende Studie umfasste schwangere Frauen in 262 gynäkologischen Praxen, bei denen zwischen Januar 2012 und Dezember 2016 eine Extrauteringravidität diagnostiziert wurde sowie schwangere Kontrollen ohne Extrauteringravidität. Die Auswirkungen der demografischen und klinischen Variablen auf das Risiko einer Extrauteringravidität wurden anhand eines multivariaten logistischen Regressionsmodells geschätzt.

**Ergebnisse:** Diese Studie umfasste 3.003 Frauen mit Extrauteringravidität und 97.194 Frauen ohne Extrauteringravidität. Das Durchschnittsalter betrug 31,4 Jahre (SA=5,9 Jahre) bei den Patientinnen mit Extrauteringravidität und 31,1 Jahre (SA=5,6 Jahre) bei den Patientinnen ohne Extrauteringravidität. Frauen Im Alter zwischen 36–40 (OR=1,12) und 41–45 Jahren (OR=1,46) hatten ein höheres Risiko einer Extrauteringravidität als Frauen im Alter zwischen 31–35 Jahren. Eine vorhergegangene Extrauteringravidität stand in engem Zusammenhang mit dem Risiko einer erneuten Extrauteringravidität (OR=8,17). Vorhergegangene Operation im Genitalbereich (OR=2,67), Endometriose (OR=1,51) und acht weitere gynäkologische Erkrankungen waren ebenfalls positiv mit Extrauteringravidität assoziiert (ORs zwischen 1,19 und 2,06). Zu guter Letzt zeigte sich bei Frauen mit einer vorherigen Diagnose einer psychiatrischen Störung ein 1,8-facher Anstieg des Risikos einer Extrauteringravidität.

**Schlussfolgerungen:** Vorhergegangene Extrauteringravidität und Operation im Genitalbereich waren eng mit Extrauteringravidität bei in deutschen gynäkologischen Praxen behandelten Frauen assoziiert. Psychiatrische Erkrankungen hatten einen zusätzlichen Einfluss auf das Risiko einer Extrauteringravidität.

## Introduction

Ectopic pregnancy is a gynecological complication that occurs in approximately 1–2% of all pregnancies and is an important cause of morbidity in women [1], [2]. A 2016 UK study estimated that post-traumatic stress disorder, depression, and anxiety are very common in women that have experienced ectopic pregnancy [3], underlying the major impact of this condition on women’s health in Western regions of the world. 

The identification of potential risk factors for the diagnosis of ectopic pregnancy has been the center of an intensive discussion in the literature in recent years [4], [5], [6], [7], [8], [9], [10]. In 2003, Bouyer and colleagues discovered that two main risk factors were infectious history and smoking status [4]. Age, prior spontaneous abortion, history of infertility, and previous use of an intrauterine device were all found to have an additional effect on the risk of ectopic pregnancy. Later, in 2006, Karaer et al. suggested in a prospective analysis of 600 women that patients with a history of ectopic pregnancy or infection of the reproductive system were more likely to develop an extra-uterine pregnancy than those free of such medical history [5]. These results were partially corroborated in 2014, when another study showed that ectopic pregnancy was associated with previous adnexal surgery, uncertain history of previous pelvic inflammatory disease, positive *Chlamydia trachomatis* IgG serology, infertility, and *in vitro* fertilization [7]. Although these previous works are of great interest, none of them was conducted in Germany, so confirming their findings in this country would be of value.

Therefore, the goal of the present retrospective study was to identify potential risk factors for ectopic pregnancy in women followed in German gynecological practices.

## Methods

### Database

This retrospective study is based on data from the Disease Analyzer database (QuintilesIMS), which compiles demographic, clinical, and pharmaceutical data obtained in an anonymous format from computer systems used in clinical practices [11]. The quality and exactness of the data (e.g., diagnoses and drug prescriptions) are regularly assessed by QuintilesIMS. Using prescription statistics for several drugs and age groups for several diagnoses, the Disease Analyzer database was found to be a representative database of clinical practices in Germany [11]. Finally, several studies focusing on gynecological disorders and using the same database have already been published [12], [13], [14].

### Study population

**Figure 1:**
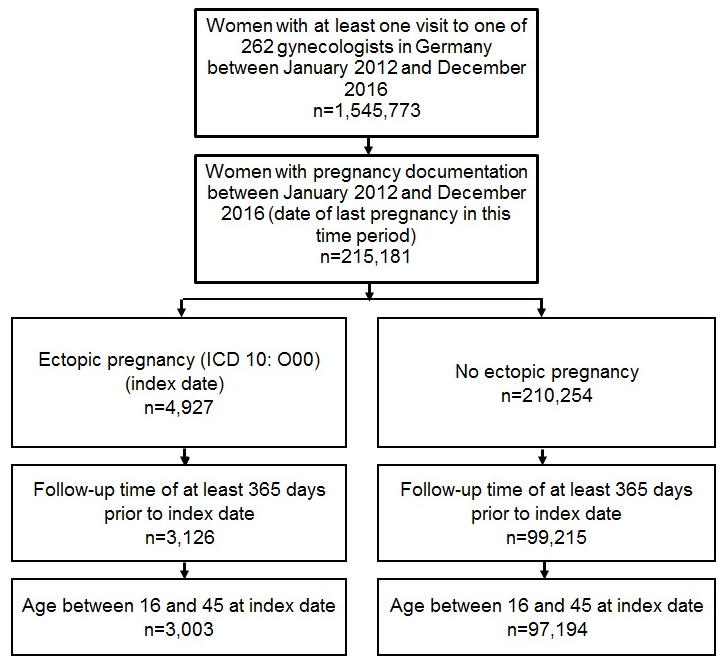
Flow chart of women included in the present retrospective case-control study

The present study included pregnant women diagnosed with ectopic pregnancy (ICD-10: O00) and pregnant women without ectopic pregnancy followed in 262 gynecological practices between January 2012 and December 2016. The index date corresponded to the date of diagnosis of ectopic or non-ectopic pregnancy. When women had more than one pregnancy during the index period (2012–2016), only the last pregnancy was considered. To be included in the analysis, patients had to be between 16 and 45 years old and followed for at least 365 prior to their index date (Figure 1 (Fig. 1)). 

### Independent variables

Demographic variables included age and type of health insurance (private or statutory), while clinical variables consisted of diagnoses documented prior to the index date. Diagnoses were only included if they were found in at least 1% of patients in either the ectopic or the non-ectopic pregnancy group. Prior ectopic pregnancy and prior genital surgery were included as co-variables if they were documented at least once in the overall medical history of the patient. Genital surgery was estimated using a combination of diagnostic documentation (“condition after surgery”) and ICD-10 codes for inflammatory or non-inflammatory diseases of female pelvic organs (N70-98). Diagnoses documented in the year prior to the index date included vulvitis (N76.2, N76.3), salpingitis, and oophoritis (N70), endometriosis (N80), erosion and ectropion of cervix uteri (N86), unspecified noninflammatory disorders of vagina (N89.9), absent, scanty, and rare menstruation (N91), excessive, frequent, and irregular menstruation (N92), abnormal uterine and vaginal bleeding (N93), mid-cycle pain (N94.0), dysmenorrhea (N94.4, N94.5, N94.6), ovarian cysts (N83.0, N83.1, N83.2), female infertility (N97), benign neoplasm of female genital organs (D25-D28), and ovarian dysfunction (E28). Finally, psychiatric diseases included depression (F32), anxiety (F41), adjustment disorder (F43), and somatoform disorder (F45).

### Statistical analyses

Descriptive analyses were obtained for all demographic and clinical variables, and the differences in patient characteristics (ectopic pregnancy versus non-ectopic pregnancy) were assessed using Wilcoxon- or Chi^2^-Test. The effects of demographical and clinical variables on the risk of developing ectopic pregnancy were estimated using a multivariate logistic regression model. P-values lower than 0.05 were considered statistically significant. All calculations were carried out using SAS 9.3 (SAS Institute, Cary, USA).

## Results

Baseline characteristics of patients are listed in Table 1 (Tab. 1). This study included 3,003 women with ectopic pregnancy and 97,194 women without ectopic pregnancy. The mean age was 31.4 years (SD=5.9 years) in ectopic pregnancy patients and 31.1 years (SD=5.6 years) in non-ectopic pregnancy patients. Factors significantly associated with the risk of being diagnosed with ectopic pregnancy are shown in Table 2 (Tab. 2). Prior to adjustment, 18 variables had a significant impact on ectopic pregnancy: age, ectopic pregnancy in the past, genital surgery in the past, vulvitis, endometriosis, erosion and ectropion of cervix uteri, noninflammatory disorders of vagina, absent, scanty and rare menstruation, excessive, frequent and irregular menstruation, abnormal uterine and vaginal bleeding, mid-cycle pain, dysmenorrhea, salpingitis and oophoritis, female infertility, benign neoplasm of female genital organs, ovarian dysfunction and psychiatric diseases (depression, anxiety, adjustment disorder and somatoform disorder). After adjustment, women aged 36–40 (OR=1.12) and 41–45 years (OR=1.46) were at a higher risk of ectopic pregnancy than women aged 31–35 years. Prior ectopic pregnancy was strongly associated with a risk of recurring ectopic pregnancy. (OR=8.17). Prior genital surgery (OR=2.67), endometriosis (OR=1.51), and eight other gynecological diseases were also positively associated with ectopic pregnancy (ORs ranging from 1.19 to 2.06). Finally, there was a 1.80-fold increase in women previously diagnosed with psychiatric disorders. When only endometriosis and dysmenorrhea were included in the regression model, both variables were significantly associated with an ectopic pregnancy diagnosis (endometriosis: OR=1.80, 95% CI: 1.26–2.57; dysmenorrhea: OR=1.50, 95% CI: 1.31–1.84). Other variables were not significantly associated with an ectopic pregnancy diagnosis (Table 3 (Tab. 3)).

## Discussion

The present German study of more than 100,000 patients showed that psychiatric disorders were associated with the risk of ectopic pregnancy. Moreover, age was positively associated with the likelihood of being diagnosed with ectopic pregnancy. Furthermore, several comorbidities, in particular prior ectopic pregnancy, prior genital surgery, and endometriosis, were found to have a significant impact on the risk of ectopic pregnancy. 

The major finding of this work is that psychiatric disorders (i.e. depression, anxiety, adjustment disorder, and somatoform disorder) favored the occurrence of ectopic pregnancies. Extra-uterine pregnancy is known to increase maternal stress, anxiety, and depression [3]. In 2016, Farren et al. showed that psychological morbidity was higher in women with miscarriage or ectopic pregnancy than in those with an ongoing pregnancy [3]. Post-traumatic stress disorder, anxiety, and depression were found in 28%, 32%, and 16%, respectively, of the pregnancy loss group one month after the end of pregnancy, whereas no women in the ongoing pregnancy group met the criteria for post-traumatic stress disorder, and only 10% met the criteria for anxiety or depression. However, no work to date has focused on the impact of psychiatric disorders or their treatments on ectopic pregnancy. To explain the present findings, we hypothesize that these diseases and their associated treatments may impair tubal function, thus leading to an impairment of the transport of the blastocyst and extra-uterine implantation. In line with this hypothesis, an animal model showed that stress exacerbates endometriosis manifestations and inflammatory parameters, potentially increasing the risk of ectopic pregnancy [15].

Another finding of this work is that previous ectopic pregnancy was a risk factor for the development of a subsequent ectopic pregnancy. In 1996, Ankum and colleagues showed in a meta-analysis of 27 case-control and 9 cohort studies that previous ectopic pregnancy was strongly associated with the risk of extra-uterine pregnancy [16]. In their 2006 analysis including 225 cases and 375 controls, Karaer et al. also estimated that the main risk factor for ectopic pregnancy was prior ectopic pregnancy (OR=13.1) [5]. That same year, in a nested case-control study conducted in the U.S. including more than 2,000 women, researchers found that the likelihood of developing ectopic pregnancy increased with the number of prior ectopic pregnancies (1 prior event: 2.98; ≥2 prior events: 16.04) [17]. These results were recently corroborated by Moini and colleagues, who discovered in 423 women followed between 2006 and 2011 that history of ectopic pregnancy was associated with a 17-fold increase in the risk of subsequent ectopic pregnancy [8]. Such findings underline the fact that recurrent ectopic pregnancies likely reflect persistence in tubal pathology and tubal dysfunction [17].

One important result of this German study is that prior genital surgery increased the likelihood of diagnosis of ectopic pregnancy. The association between previous genital surgery and ectopic pregnancy has been the center of intensive research since the beginning of the 1990s. In that same 1996 meta-analysis mentioned above, it was discovered that genital surgery led to a major increase in the risk of ectopic pregnancy (OR=4.7) [16]. A more recent study estimated that women with tubal damage were 2.5–3 times more likely to be affected by ectopic pregnancy when compared to controls, although whether the increased risk was explained by the genital surgery or by the underlying disorder remained uncertain [8]. Although tubal surgery is a likely risk factor for ectopic pregnancy, the impact of nontubal surgery remains unclear. One of the first studies to focus on this matter found no significant association between abdominal or pelvic surgery and ectopic pregnancy [18]. By contrast, other works showed that such surgery increased the likelihood of developing this pregnancy-related complication [19], [20]. More recently, in 2006, Barnhart and colleagues estimated in women followed in Pennsylvania that prior nontubal pelvic surgery demonstrated no association with ectopic pregnancy [17]. Since we could not distinguish tubal from nontubal surgeries in the present retrospective study, their respective impacts were not analyzed separately. Moreover, erosion and ectropion of cervix uteri were found to be additional risk factors for ectopic pregnancy. This finding may also indirectly reflect the relationship between local treatment (either surgical or conservative) and possibly occult ascending infections disturbing tubal function [21], [22].

 We further showed that endometriosis was a risk factor for ectopic pregnancy. In 2006, Clayton and colleagues discovered in 94,118 pregnancies with assisted reproductive technology procedures that endometriosis led to a 1.3-fold increase in the risk of ectopic pregnancy [23]. It was later estimated in a cohort of 14,655 women followed up over a 30-year period (1981–2010) that individuals affected by this chronic gynecological disorder were at a higher risk of miscarriage (OR=1.76) and ectopic pregnancy (OR=2.70) than those free of this condition [24]. The hypothesis is that, when endometrial-like tissue adheres to the ovarian tubes, it can disturb tubal permeability and blastocyst transport [25]. Additionally, it was found in our work that dysmenorrhea, a symptom frequently correlated with endometriosis [26], was associated with a 1.35-fold increase in the risk of ectopic pregnancy. This finding suggests that dysmenorrhea is an independent risk factor for the development of an ectopic pregnancy. Nonetheless, it is also possible that dysmenorrhea, which is found in a wide range of conditions, only reflects the significant impact of variables not included in the present logistic regression analysis on the odd of being diagnosed with an ectopic pregnancy.

Women diagnosed with vulvitis were further found to be at a particular risk of developing ectopic pregnancy compared to those without this condition. In their 2003 study, Bouyer et al. showed that a history of genital infection was associated with a 3.4-fold increase in the risk of being affected by ectopic pregnancy [4]. When infectious history and prior tubal surgery were considered together, they accounted for approximately 33% of ectopic pregnancies. These findings were later corroborated by Karaer and colleagues [5], as they found prior infection of the reproductive system to be the second most important factor for the diagnosis of extra-uterine pregnancy. Among all pathogens, *Chlamydia trachomatis* plays a major role in the epidemiology of ectopic pregnancy. In 2007, Bakken et al. discovered in a Norwegian study of 616 cases and 1,848 controls that *Chlamydia trachomatis* infection was associated with an elevated ectopic pregnancy risk [27]. That same analysis further estimated that such association was only significant in the youngest group of women. More recently, in 2014, Li et al. found in 1,789 individuals that the risk of ectopic pregnancy was notably associated with a positive IgG serology for *Chlamydia trachomatis* [7]. Although they observed a positive relationship between vulvitis and ectopic pregnancy in this retrospective analysis, salpingitis and oophoritis had no significant impact on such complication. As salpingitis and oophoritis were only found in 1.0% of cases and 0.4% of controls, it is likely that this absence of a significant result is explained by the present study’s lack of power in this regard.

Menstrual dysregulation, vaginal bleeding, and mid-cycle pain were additional risk factors for ectopic pregnancy. In 2016, Ayim and colleagues discovered in 1,320 women followed in the UK that pelvic pain (OR=2.4) and diarrhea (OR=2.2) in the 24 hours prior to their arrival at the early pregnancy assessment unit increased the risk of ectopic pregnancy [28]. Interestingly, such risk increased by 20% for every one-day increment in duration of vaginal bleeding. This piece of data underlines the need to consider any women with bleeding and/or pain in the early stages of pregnancy as having a potential extra-uterine pregnancy. These women should undergo an ultrasound scan [28]. 

Finally, maternal age increased the risk of developing ectopic pregnancy. Findings have conflicted over the past decades regarding this association. In the beginning of the 1990s, Coste and colleagues conducted a case-control study in seven Paris-area maternity hospitals and analyzed the risk factors for ectopic pregnancy [29]. Although women aged 30–34, 35–39 and ≥40 years were more likely to be diagnosed with ectopic pregnancy than those aged 20–24 years in the univariate logistic regression model, the multivariate analysis showed that age was not significantly associated with the risk of ectopic pregnancy. More recently, in 2006, Karaer et al. observed that the risk of ectopic pregnancy increased progressively with maternal age [5]. In line with these results, Parashi and colleagues discovered no significant relationship between the two variables after controlling for several factors in 150 women with ectopic pregnancy and 300 controls from Iran [9]. By contrast, in 2003, another French study including 2,486 women showed that age led to an increase in the likelihood of being diagnosed with this gynecological complication [4]. The hypothesis to explain such a result is that age involves major changes in tubal function, indirectly predisposing women to an extra-uterine pregnancy [30]. 

Retrospective primary care database analyses are generally limited by the validity and completeness of the data on which they are based. The present study included several limitations, such as the assessment of ectopic pregnancy and co-morbidities, which relied solely on ICD codes entered by gynecologists. As a result, some detailed diagnosis codes (ICD 10 code level 4) were not available and only unspecified codes were used (ICD code level 3). Furthermore, data pertaining to socioeconomic status (e.g., education and income) and lifestyle-related risk factors (e.g., smoking, alcohol, and physical activity) were also lacking. The study also had several strengths. More than 100,000 women in numerous gynecological practices were included in this study. Another strength was the use of ‘real-world’ data in gynecological practices where diagnoses were continuously documented, allowing for unbiased exposure assessment (no recall bias).

Prior ectopic pregnancy and prior genital surgery were strongly associated with ectopic pregnancy in women followed in German gynecological practices. Psychiatric disorders displayed an additional effect on the risk of ectopic pregnancy. Further studies are needed to gain a better understanding of the potential impact of other conditions on the risk of ectopic pregnancy. 

## Data

Data for this article are available from the Dryad Repository: http://dx.doi.org/10.5061/dryad.pv805 [31].

## Notes

### Competing interests

Karel Kostev is an employee of Quintiles IMS, which now is part of IQVIA. IQVIA (https://www.iqvia.com/about-us) is a contract research organization providing information, services, and technology for the healthcare industry. LJ, MK, and KK declare that they have no potential conflicts of interest with respect to the research, authorship, and/or publication of this article.

### Acknowledgments

Professional English language editing services were provided by Claudia Jones, MA, Radford, Virginia, United States.

## Figures and Tables

**Table 1:**
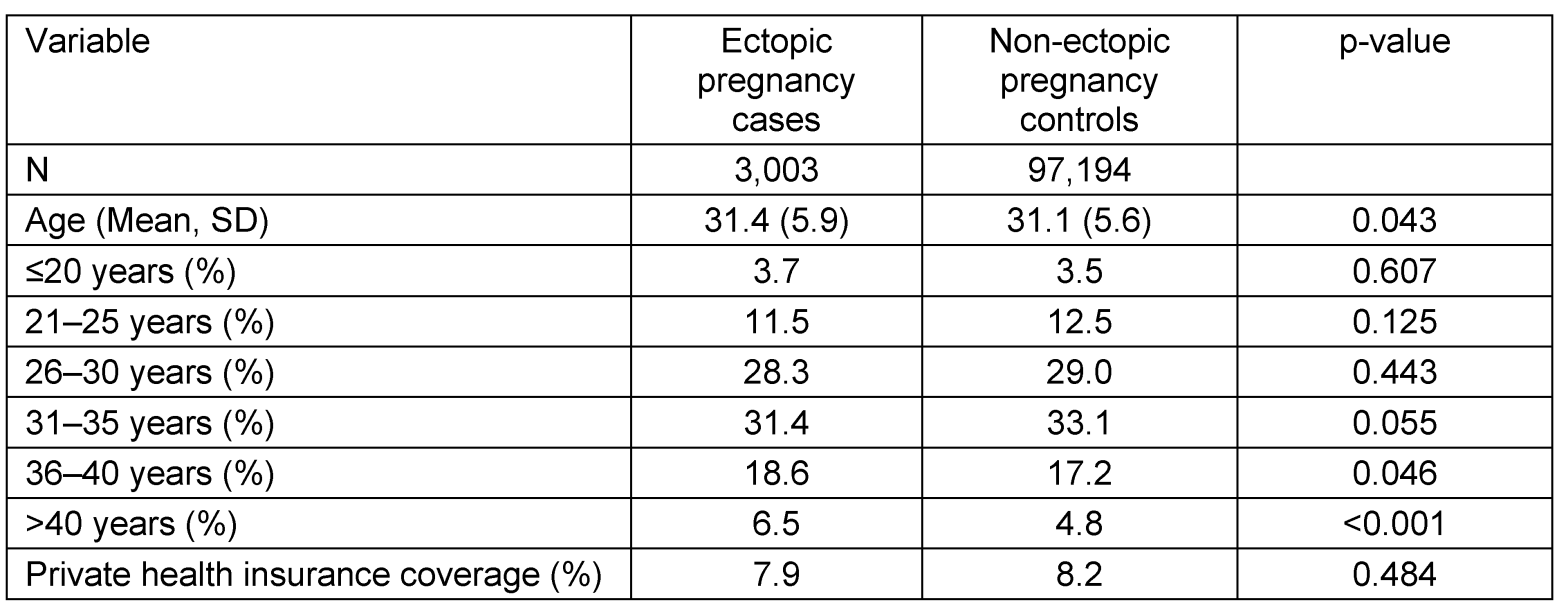
Characteristics of women with or without ectopic pregnancy (QuintilesIMS Disease Analyzer database)

**Table 2:**
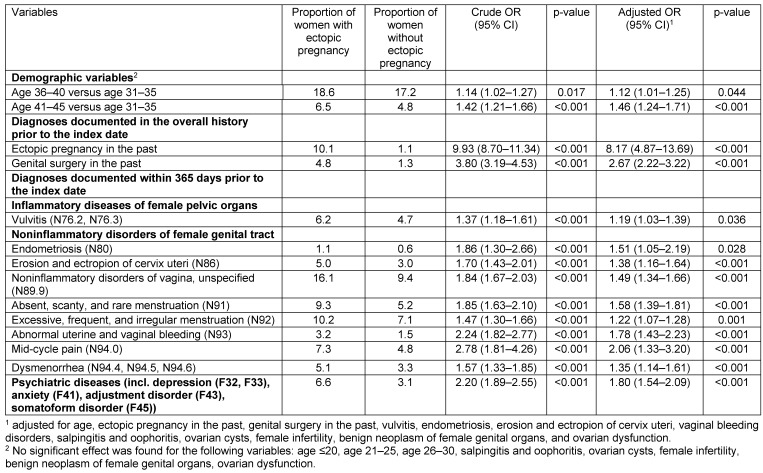
Factors significantly associated with an ectopic pregnancy diagnosis in women treated in gynecological practices (logistic regression model)

**Table 3:**
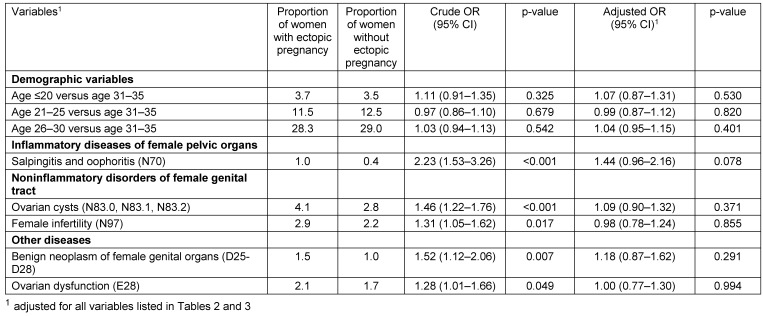
Factors not significantly associated with an ectopic pregnancy diagnosis in women treated in gynecological practices (logistic regression model)
